# An HDAC-dependent epigenetic mechanism that enhances the efficacy of the antidepressant drug fluoxetine

**DOI:** 10.1038/srep08171

**Published:** 2015-02-02

**Authors:** C. Schmauss

**Affiliations:** 1 Department of Psychiatry, Columbia University, New York, NY 10032; 2 Division of Molecular Therapeutics, New York State Psychiatric Institute, New York, NY 10032

## Abstract

Depression is a prevalent and debilitating psychiatric illnesses. However, currently prescribed antidepressant drugs are only efficacious in a limited group of patients. Studies on Balb/c mice suggested that histone deacetylase (HDAC) inhibition may enhance the efficacy of the widely-prescribed antidepressant drug fluoxetine. This study shows that reducing HDAC activity in fluoxetine-treated Balb/c mice leads to robust antidepressant and anxiolytic effects. While reducing the activity of class I HDACs 1 and 3 led to antidepressant effects, additional class II HDAC inhibition was necessary to exert anxiolytic effects. In fluoxetine-treated mice, HDAC inhibitors increased enrichment of acetylated histone H4 protein and RNA polymerase II at promotor 3 of the brain-derived neurotrophic factor (Bdnf) gene and increased Bdnf transcription from this promotor. Reducing Bdnf-stimulated tropomyosin kinase B receptor activation in fluoxetine-treated mice with low HDAC activity abolished the behavioral effects of fluoxetine, suggesting that the HDAC-triggered epigenetic stimulation of Bdnf expression is critical for therapeutic efficacy.

Mood disorders (major depressive disorders (MDD), bipolar disorder) are the most prevalent among all psychiatric illnesses and they are the second leading cause of disability worldwide^1^,^2^. It is estimated that the overall lifetime risk for MDD in the USA is ~16%^3^. The pharmacological treatment of mood disorders is predominantly monoamine-based. Commonly prescribed drugs are tricyclic antidepressants, monoamine oxidase inhibitors, or selective serotonin-reuptake inhibitors (SSRIs). These drugs elicit antidepressant effects only after long-term treatment^4^, and many of them have considerable side effects^5^. Moreover, recent data illustrate that currently prescribed antidepressant drugs are only efficacious in a limited group of patients (moderate to severe, but not mild, depression^6^,^7^,^8^), and that a history of early life stress renders depressed patients particularly insensitive to antidepressant treatment^9^,^10^.

In addition to genetic polymorphisms that could influence the outcome of treatment^11^, it is possible that epigenetic mechanisms modulate treatment response^12^. Indeed, a recent study on inbred strains of mice discovered adaptive epigenetic responses to early life stress that ameliorated the severity of the adult emotional psychopathology and also enhanced the response to antidepressant treatment with the SSRI fluoxetine^13^. Specifically, after early life stress exposure, the stress-susceptible inbred mouse strain Balb/c develops decreased activity of several HDACs that, in turn, leads to increased levels of acetylated histone H4 protein, especially acH4K12. These epigenetic marks are established by mid-adolescence, and they persist into adulthood. While blunting this adaptive response by reducing the expression of acH4K12 during adolescent development further impaired the adult emotional phenotype, adolescent fluoxetine treatment elevated the expression of acetylated histone H4 proteins even further and, importantly, reduced depressive behavior^13^. This finding led to the hypothesis that histone H4 acetylation is a critical determinant of the antidepressant efficacy of fluoxetine, a hypothesis tested in the present study on Balb/c mice co-treated with various HDAC inhibitors and fluoxetine during adolescence or adulthood. The results show that the combined treatment with fluoxetine and various HDAC inhibitors led to significantly enhanced enrichment of acH4K12 at the Bdnf gene promotor 3 and increased expression of Bdnf transcript variant 3. Moreover, treatment with fluoxetine and a class I HDAC inhibitor elicited pronounced antidepressant effects, while additional inhibition of class II HDACs was required to also achieve significant anxiolytic effects. These data illustrate that HDAC inhibitors can significantly enhance the therapeutic effects of fluoxetine.

## Results

### Epigenetic and behavioral effects of adolescent fluoxetine in mice exposed to early life stress

Balb/c mice exposed to infant maternal separation (IMS) during postnatal ages P2 to P15 (here referred to as IMS mice) exhibit reduced activity of class I HDACs 1, 3, and 8 and class II HDACs 7 and 10 in the forebrain neocortex (but not in striatum or hippocampus)^13^. This leads to a persistently increased acetylation of histone H4 protein, especially acH4K12^13^. Since adolescent fluoxetine treatment of IMS Balb/c mice further increased their levels of acH4K12 and exerted robust antidepressant effects, the present study examined the functional link between the fluoxetine-triggered increased acetylation of H4K12 in IMS mice and the observed effect of this drug on the emotional phenotype.

As an epigenetic mark of active gene expression, acH4K12 exerts gene-specific effects on transcription rates in IMS Balb/c mice^14^. Since the neurotrophic factor Bdnf is a critical mediator of activity-dependent plasticity in the developing and mature brain, and since changes in neuronal plasticity and Bdnf signaling have been implicated both in the etiology of depression and antidepressant drug action^15^, the present study first examined whether increased acetylation of histone H4K12 also affects transcription of the Bdnf gene.

The rodent Bdnf gene contains multiple promotors that generate transcripts with different 5′ exons spliced to a common 3′ exon encoding the mature part of the Bdnf protein^16^, thus allowing multiple points of activity-dependent Bdnf mRNA regulation^17^. Hence, chromatin immunoprecipitation (ChIP) experiments were performed that targeted 5 promotors of the mouse Bdnf gene using antibodies against acH4K12 and the actively elongating form of RNA polymerase II (Pol II). Results obtained from forebrain neocortical tissues of non-treated IMS mice and IMS mice treated with fluoxetine during adolescence were compared to corresponding results obtained from non-stressed, standard-facility-reared (SFR) controls.

**Figure 1:**
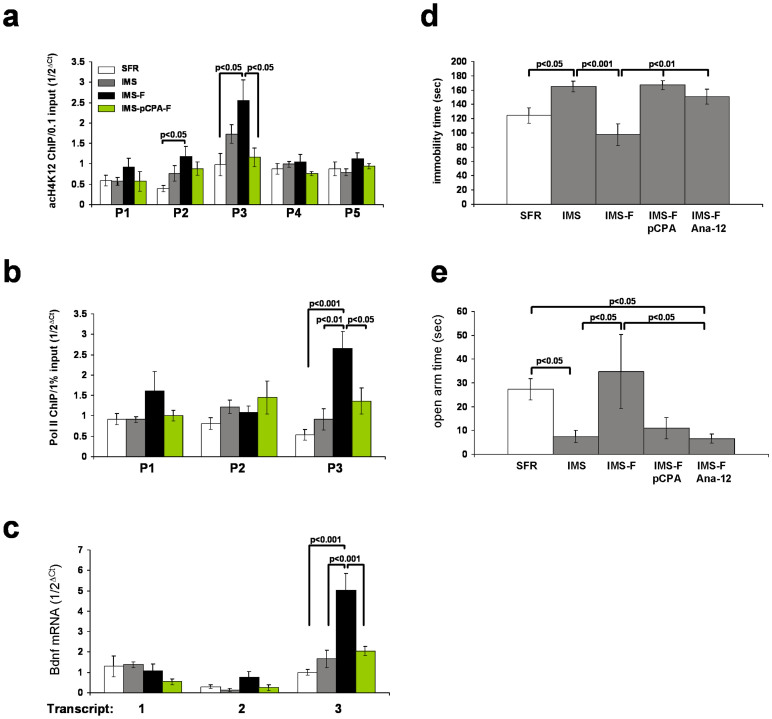
Adolescent fluoxetine potentiates the enrichment of acH4K12 histone and RNA Polymerase II at Bdnf promotor 3 and increases Bdnf transcript variant 3 expression in the forebrain neocortex of IMS mice. (a) acH4K12 ChIP targeting Bdnf promoters 1 to 5. (b) Pol II ChIP targeting promoters 1 to 3. (c) Real-time PCR measures of Bdnf transcript variants 1 to 3. Data (mean ± sem of 7 animals/group) are from SFR and IMS mice, and IMS mice treated with fluoxetine during adolescence in the absence (IMS-F) or presence of pCPA (IMS-pCPA-F). Data were compared by ANOVA (acH4K12 ChIP: P1: F(_3,27_) = 0.94, p = 0.44; P2:F(_3,27_) = 3.53, p = 0.03; P3: F(_3,27_) = 4.46, p = 0.013; P4: F(_3,27_) = 0.853, p = 0.48; P5: F(_3,27_) = 1.19, p = 0.335; Pol II ChIP: P1: F(_3,27_) = 1.78, p = 0.18; P2: F(_3,27_) = 2.26, p = 0.11; P3: F_(3,27_) = 9.37 p = 0.0003); Bdnf mRNA: P1: F(_3,27_) = 1.15, p = 0.35; P2: F(_3,27_) = 2.47, p = 0.1; P3: F(_3,27_) = 16.04, p = 0.0001)) and statistical differences were resolved post hoc (Tukey Kramer multiple comparisons) as indicated. (d) Behavior of IMS, fluoxetine-treated IMS mice, and fluoxetine-treated IMS mice co-treated with pCPA (IMS-F pCPA) or Ana-12 (IMS-F Ana12) in the Forced Swim Test (FST). (e) Behavior of the same groups of mice in the Elevated Plus Maze (EPM). Data are mean ± sem of 7 animals/group. Significant differences revealed by ANOVA (FST: F(_4,34_) = 7.64, p = 0.0003; EPM: F(_4,34_) = 5.76, p = 0.0014) were resolved post hoc (Tukey Kramer multiple comparisons) as indicated.

As shown in Fig. 1a, levels of acH4K12 at Bdnf promotors 2 and 3 were elevated in IMS mice, but compared to SFR controls, this enrichment did not reach statistical significance. In fluoxetine-treated IMS Balb/c mice, however, enrichment of acH4K12 at promotors 2 and 3 was further elevated and differed significantly from SFR controls. Neither IMS exposure nor fluoxetine treatment of IMS Balb/c mice affected their levels of acH4K12 associated with Bdnf promotors 1, 4, and 5 (Fig. 1a).

Corresponding Pol II ChIPs revealed that, in fluoxetine-treated IMS Balb/c mice, the density of Pol II was significantly higher only at Bdnf promotor 3 (Fig. 1b), a finding consistent with results of real-time RT-PCR measures which revealed significantly increased expression of Bdnf mRNA transcript variant 3 (but not transcript variants 1 and 2) (Fig. 1c).

Of note, contrary to studies on adult rats exposed to early life stress that showed decreased Bdnf expression in the hippocampus and/or prefrontal cortex^18^,^19^, in IMS Balb/c mice Bdnf mRNA expression does not significantly differ from SFR controls. However, IMS Balb/c mice have been shown to uniquely develop a histone-based epigenetic response to early life stress that not only ameliorates the severity of the emotional psychopathology in adulthood^13^, but also elevates the level of acH4K12 at Bdnf promotors 2 and 3 (Fig.1a). Although this increased acH4K12 enrichment does not reach significance, it is conceivable that it contributes to maintaining normal Bdnf mRNA expression levels in these mice.

Since fluoxetine is an antidepressant drug of the SSRI class, additional studies examined whether its effect on Bdnf gene transcription is dependent upon elevated levels of extracellular serotonin (5-HT). Hence, IMS Balb/c mice were depleted of 5-HT during adolescent fluoxetine treatment by co-treating them with the irreversible tryptophan hydroxylase inhibitor para-chlorophenylalaninin (pCPA; see Methods). As shown in Fig. 1a–c, in 5-HT depleted mice, the effect of fluoxetine on acH4K12 and Pol II enrichment was abolished, and the expression of Bdnf transcript variant 3 mRNA was also no longer increased.

5-HT depletion also abolished the antidepressant effects of adolescent fluoxetine treatment in IMS mice. Contrary to fluoxetine-treated IMS mice that exhibit significantly reduced depression-like behavior in the forced swim test (FST; Fig. 1d) and reduced anxiety-like behavior in the Elevated Plus Maze (EPM; Fig. 1e), the FST and EPM behaviors of fluoxetine-treated IMS mice that were depleted of 5-HT did not differ from the corresponding behavioral phenotypes of non-treated IMS mice (Fig. 1d–e).

Mature Bdnf mediates its effect through tropomyosin kinase B (TrkB) receptors that are abundantly expressed in the frontal cortex^20^. To test whether increased Bdnf-TrkB signaling mediates the behavioral effect of fluoxetine, the effect of the low molecular weight TrkB inhibitor Ana-12^21^ was investigated in IMS Balb/c treated with fluoxetine during adolescence. It is important to note that, contrary to the previously reported anxiolytic effects (in EPM and the novelty-suppressed feeding tests) and antidepressant effects (in the FST and tail suspension test) after acute Ana-12 treatment of adult F1 hybrid C57Bl/6 and 129SveV mice^21^, adolescent Ana-12 treatment of SFR Balb/c mice did not alter the behavior in the EPM and only moderately decreased the immobility measures of the FST (Supplementary Fig. 1). In fluoxetine-treated IMS Balb/c mice, however, Ana-12 co-treatment abolished the behavioral effects of fluoxetine in the FST and EPM (Fig. 1d–e).

In conclusion, in IMS Balb/c mice, fluoxetine's effects on emotional behavior are triggered by a serotonin-dependent epigenetic mechanism involving an acH4K12-triggered increase in the expression of Bdnf transcript variant 3 and increased Bdnf-TrkB receptor activation.

### Effects of adolescent fluoxetine/HDAC inhibitor co-treatment in non-stressed Balb/c mice

**Figure 2:**
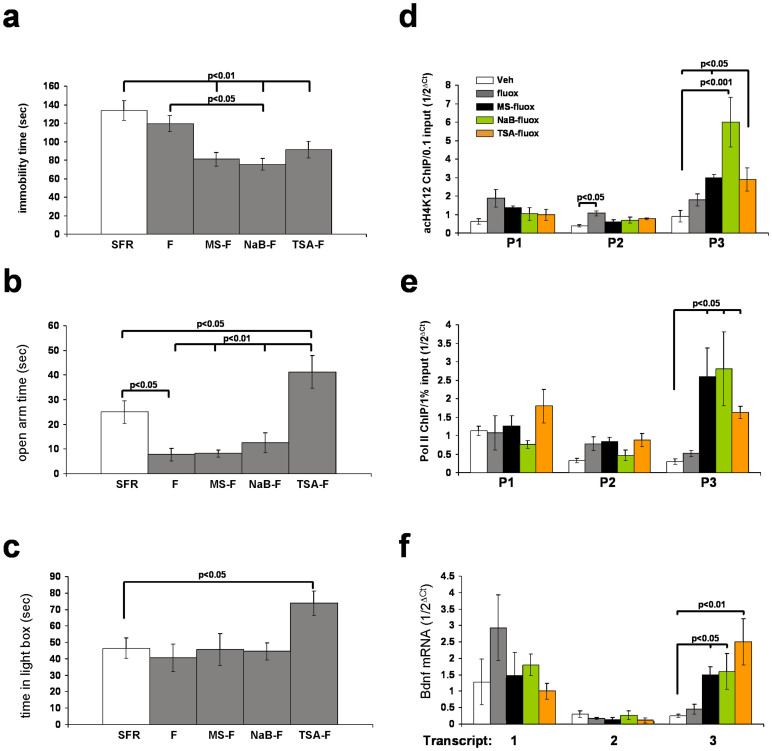
HDAC inhibitors enhance the effects of adolescent fluoxetine in SFR mice. (a) Behavior of non-treated SFR mice, SFR mice treated with fluoxetine only (F) or co-treated with MS-275 (MS-F), NaB (NaB-F), or TSA (TSA-F) in the FST. (b) Behavior of the same groups of mice in the EPM. (c) Behavior in the Light/Dark Exploration test (L/D test). Statistical differences were determined using ANOVA (FST: F(_4,40_) = 4.902, p = 0.0025 (n = 8/group); EPM: F(_4,35_) = 7.296, p = 0.0003 (n = 7/group); L/D test: F(_4,35_) = 3.119, p = 0.03 (n = 7/group)) and resolved *post hoc* (Tukey Kramer multiple comparisons) as indicated. (d) Enrichment of acH4K12 at Bdnf promotors 1 to 3. (e) Enrichment of RNA Polymerase II at Bdnf promotors 1 to 3. (f) Real-time RT-PCR measures of Bdnf transcript variants 1 to 3. Data in d–f were obtained from forebrain neocortex of SFR mice treated with fluoxetine alone or co-treated with MS-275, NaB, or TSA during adolescence and they were compared to corresponding data obtained from non-treated SFR mice that are also shown in Fig. 1. Significant differences revealed by ANOVA (acH4K12 ChIP: P1 F(_4,23_) = 2.587,p = 0.07; P2 F(_4,20_) = 5.104, p = 0.0085; P3 F(_4,21_) = 7.565, p = 0.001; Pol II ChIP: P1 F(_4,18_) = 1.784,p = 0.1882; P2 F(_4,20_) = 2.515, p = 0.0854; P3 F(_4,25_) = 5.803, p = 0.0026)) were resolve *post hoc* as indicated. For real-time RT-PCR measures of Bdnf mRNA levels, Kruskal Wallis non-parametric ANOVA revealed significant differences that were resolved using Dunn's multiple comparison tests as indicated.

In SFR control Balb/c mice, adolescent fluoxetine treatment did not affect the behavior in the FST (Fig. 2a) and, consistent with previous reports^22^,^23^, it paradoxically exerted anxiogenic effects in the EPM (Fig. 2b). This anxiogenic effect, however, was not detected in an additional test of anxiety-like behavior, the Light/Dark exploration test (L/D test; Fig. 2c). The following experiments tested whether reducing the HDAC activity in SFR mice would render adolescent fluoxetine treatment effective.

A first experiment focused on class I HDACs 1 and 3 for which MS-275 is a selective inhibitor^24^. As shown in Fig. 2a, in SFR control mice, adolescent co-treatment with MS-275 and fluoxetine led to significantly improved behavior in the FST. Contrary to this antidepressant effect, this co-treatment did not affect the anxiety-like phenotypes measured in the EPM and L/D test (Fig. 2b–c). A next experiment replaced MS-275 with sodium butyrate (NaB) that, in addition to HDAC 1 and 3 also inhibits class II HDAC 7. Like MS-275/fluoxetine co-treatment, NaB/fluoxetine treatment improved the FST behavior but left the behavioral phenotypes measures in the EPM and L/D test unaltered. Finally, trichostatin A (TSA) was co-administered with fluoxetine during adolescence. TSA has the broadest class I/II HDAC inhibitor profile. It inhibits HDACs 1, 3, 4, 6, and 10 but has no effects on HDAC 8. In SFR mice, only this co-treatment exerted both antidepressant effects in the FST and anxiolytic effects in the EPM and L/D test (Fig. 1a–c).

In fluoxetine-treated SFR mice, the effects of the HDAC inhibitor (HDACi) co-treatment on the emotional behavior are not due to the effects of either of the HDACi alone. After adolescent treatment with the HDACis, neither of them significantly altered the behavior in the FST, EPM and L/D test (Supplementary Fig. 2).

Similar to results obtained from IMS mice, co-treatment of SFR mice with fluoxetine and an HDACi during adolescence influenced the epigenetic marks at Bdnf promotor 3. While fluoxetine treatment alone led to increased enrichment of acH4K12 at Bdnf promotor 2, in all groups of fluoxetine-treated mice co-treated with an HDACi enrichment of acH4K12 was only increased at Bdnf promotor 3 (Fig. 2d). Moreover, increased density of Pol II was only detected at Bdnf promotor 3, and this increase reached significance only in co-treated animals, but not in animals that only received fluoxetine (Fig. 2e). Consistent with results obtained for the Pol II ChIP, only Bdnf transcript variant 3 mRNA was increased in co-treated animals, with the largest increase detected in fluoxetine/TSA-treated animals (Fig. 2f). The results suggest that increased Bdnf transcript variant 3 expression is critical for the enhancement of fluoxetine efficacy by HDACis.

Adolescent treatment with HDACis alone did not significantly raise the levels of acH4K12 at Bdnf promotors 1 to 3 in SFR mice (Supplementary Fig. 3). Moreover, although the density of Pol II was higher at Bdnf promotors 2 and 3, this increase did not reach significance with the exception of NaB treatment (which significantly increased the density of Pol II at Bdnf promotor 3 when compared with SFR controls (Supplementary Fig. 3).

In summary, in both SFR and IMS Balb/c mice, the therapeutic efficacy of adolescent fluoxetine treatment is enhanced when HDAC activity is reduced. Studies with SFR mice showed that, while inhibition of class I HDACs 1 and 3 is sufficient to enhance the antidepressant efficacy of fluoxetine, additional inhibition of class II HDACs is required to also increase the anxiolytic effects.

### Effects of adult fluoxetine/HDACi co-treatment in non-stressed SFR mice

**Figure 3:**
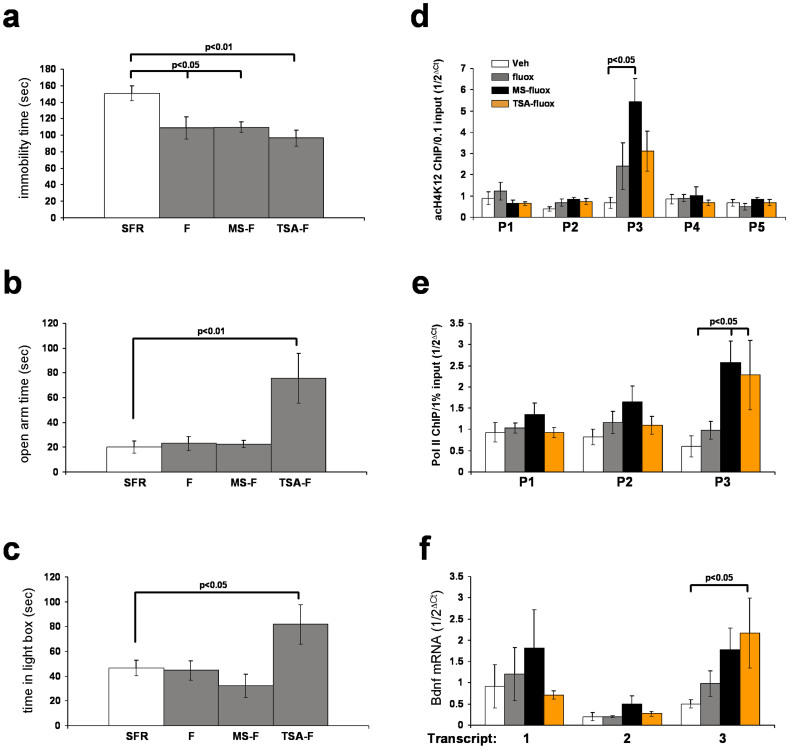
The effects of adult fluoxetine in SFR mice in the presence and absence of HDAC inhibitors. (a) Behavior of non-treated SFR mice, SFR mice treated with fluoxetine only (F) or co-treated with MS-275 (MS-F) or TSA (TSA-F) in the FST. (b) Corresponding EPM and (c) L/D test behavior. Statistical differences were determined using ANOVA (FST: F(_3,32_) = 5.684, p = 0.0033 (n = 8/group); EPM: F(_3,32_) = 6.689, p = 0.0016 (n = 8/group); L/D test: F(_3,26_) = 3.119, p = 0.03 (n = 7–8/group)) and resolved *post hoc* (Tukey Kramer multiple comparisons) as indicated. (d) The effects of adult fluoxetine on acH4K12 enrichment at promotors 1 to 5 the Bdnf gene in the presence and absence of HDAC inhibitors. (e) Corresponding Pol II enrichment at promotors 1 to 3 and (f), Bdnf transcript variants 1 to 3 mRNA expression. Data are mean ± sem of measures from 5–6 animals per group. ANOVA revealed significant differences for acH4K12 levels only at Bdnf promotor 3 (F(_3, 22_) = 4.328, p = 0.0174) that were resolved *post hoc* as indicated. For the Pol II densities, significant differences revealed by ANOVA (F(_3,23_) = 4.965, p0.0098) were found only for Bdnf promotor 3, and *post hoc* Tukey Kramer multiple comparisons resolved these differences for fluoxetine-treated mice that were co-treated with MS-275 or TSA. For real-time RT-PCR measures of Bdnf transcript variant 1 to 3 expression, ANOVA revealed no significant differences in expression of transcript variants 1 (p = 0.25) and 2 (p = 0.07) between groups, but significant differences revealed for transcript variant 3 (F(_3,20_) = 3.19, p = 0.0049)) were resolved *post hoc* as indicated.

The next experiments tested whether HDACis also enhance the therapeutic efficacy of fluoxetine in adulthood. These experiments focused on the class I HDAC inhibitor MS-275 and the class I/II HDAC inhibitor TSA. As shown in Fig. 3a, although adolescent fluoxetine had no effect on FST behavior of SRF mice, adult fluoxetine treatment of SFR mice exerted significant antidepressant effects in this test, and neither MS-275 nor TSA co-treatment enhanced this effect. Similar to results in adolescent animals, treatment of adult SFR mice with fluoxetine alone (or with MS-275) did not affect their behavior in the EPM (Fig. 3b) and L/D tests (Fig. 3c), but co-treatment with TSA led to robust anxiolytic effects (Fig. 3b–c).

Fig. 3d summarizes the results of acH4K12 ChIP experiments targeting Bdnf promotors 1 to 5 in SFR mice treated in adulthood with fluoxetine in the presence or absence of either MS-275 or TSA. Neither treatment altered the enrichment of acH4K12 at Bdnf promotors 1, 2, 4, and 5. At Bdnf promotor 3, however, the levels of acH4K12 were elevated after fluoxetine treatment, and further elevated after co-treatment with MS-275 and TSA. While the increased enrichment of acH4K12 did not quite reach significance after fluoxetine mono-treatment and after fluoxetine TSA co-treatment, the enrichment measured after fluoxetine/MS-275 co-treatment was significant. Similar to results obtained after adolescent treatment, Pol II ChIPs further revealed that co-treatment with either MS-275 or TSA led to significantly increased densities of Pol II at Bdnf promotor 3 (Fig. 3e). Finally, although fluoxetine mono-treatment and co-treatment with MS-275 and TSA led to elevated levels of Bdnf transcript variant 3 mRNA, the highest mRNA levels were expressed in fluoxetine/TSA co-treated mice, and only this increase differed significantly from vehicle-treated controls (Fig. 3f).

In conclusion, HDAC inhibition during adult fluoxetine treatment exerts effects similar to those observed after adolescent treatment.

## Discussion

The highly elevated emotional phenotype of the inbred strain Balb/c mice at baseline and after stress exposure makes this mouse strain ideal for testing the efficacy of antidepressant drugs. The present study shows however, that contrary to the robust antidepressant and anxiolytic effects of adolescent fluoxetine in Balb/c mice with a history of early life stress (IMS mice), adolescent fluoxetine exerts no effects on the emotional behavior of non-stressed Balb/c mice (SFR mice). This lack of responsiveness to adolescent fluoxetine has also been detected in previous studies^25^,^26^. Here, the mechanisms underlying the robust antidepressant and anxiolytic effects of adolescent fluoxetine in IMS mice were investigated with a focus on the role of reduced HDAC activity that is characteristic for IMS Balb/c mice where it triggers a persistent increase in acetylation of histone H4K12, an epigenetic mark that is further elevated after adolescent fluoxetine treatment^13^. Indeed, the present study shows that adolescent fluoxetine treatment of IMS Balb/c mice leads to a promotor-specific increase of acH4K12 and Pol II association with the Bdnf gene, ultimately leading to increased transcription of Bdnf mRNA transcript variant 3. (Of note, the present study uses the original Bdnf promotor numbering system^16^. In the revised nomenclature^27^, the original promotor 3 would be numbered promotor 4). This finding is of particular interest because changes in Bdnf-TrkB signaling have been implicated in both the etiology of depression and antidepressant drug action^15^, and co-treatment of IMS Balb/c mice with fluoxetine and the TrkB receptor blocker Ana-12 did indeed abolish the effects of fluoxetine on emotional behavior. Moreover, in IMS Balb/c mice that were depleted of serotonin during fluoxetine treatment, the effects of fluoxetine on emotional behavior and the fluoxetine-triggered Bdnf-specific epigenetic phenotype were completely abolished. Hence, fluoxetine's effects in these mice are mediated by endogenous serotonin and may thus be replicable with other SSRIs.

In IMS Balb/c mice, increased H4K12 acetylation is due to decreased HDAC activity^13^, suggesting that the difference in HDAC activity between SFR and IMS Balb/c mice accounts for the difference in responsiveness to adolescent fluoxetine treatment. Indeed, when fluoxetine-treated SFR mice were co-treated with HDAC inhibitors that target the HDACs affected by IMS exposure, acH4K12 and Pol II were significantly enriched at Bdnf promotor 3, and the expression of Bdnf transcript variant 3 (not significantly altered by fluoxetine mono-treatment) was significantly increased. Interestingly, earlier studies found the same promotor 3 of the Bdnf gene specifically responsible for membrane-depolarization induced increase in Bdnf expression in cultured cortical neurons (where transcriptional activation at promotor 3 accounts for most of the Bdnf expression)^28^. Of note, although the largest increase in Bdnf mRNA expression was found in fluoxetine-treated mice that were co-treated with the most broadly acting class I/II HDAC inhibitor TSA, these mRNA levels do not significantly differ when compared with fluoxetine-treated SFR mice co-treated with MS-275 or NaB.

In addition to the epigenetic phenotype of the co-treated SFR mice that strongly resembles that of fluoxetine-treated IMS Balb/c mice, the therapeutic efficacy fluoxetine was also significantly enhanced in these co-treated SFR mice. However, different HDAC inhibitors exerted different effects on emotional behavior. While inhibition of class I HDACs 1 and 3 was sufficient to enhance the antidepressant efficacy of fluoxetine (both HDACs are affected by IMS exposure), additional HDAC inhibition was required to restore the anxiolytic effects observed in fluoxetine-treated IMS mice. This was accomplished by TSA treatment that also enhanced the anxiolytic effects of fluoxetine, an effect most likely due to TSA's ability to inhibit class II HDAC 10 which is also affected by IMS exposure^13^. Of note, the remaining HDACs affected by IMS exposure are class II HDAC 7 and class I HDAC 8^13^. However, TSA does not inhibit HDAC 8 and NaB, which also inhibits HDAC7, did not promote the anxiolytic effects of fluoxetine in SFR mice. Hence, a role for HDACs 7 and 8 in modulating the responsiveness to fluoxetine treatment is not likely. It is, however, entirely possible that the effects of TSA co-treatment on anxiety-like phenotypes are not only due to increased Bdnf expression, especially since Bdnf expression levels do not significantly differ between MS-275, NaB, or TSA co-treated mice (see Fig.2c), and that the anxiolytic effects triggered by TSA/fluoxetine co-treatment are also mediated by changes in expression of other (yet to be identified) genes that play a role in the expression of anxiety-related phenotypes and whose expression is modulated by the HDACs targeted by TSA. Nevertheless, the differential enhancement by HDAC inhibitors of fluoxetine's effects on depression and anxiety observed in the present study is noteworthy because, despite a high rate of co-morbidity, depression and anxiety are clinically distinct psychiatric disorders and may require treatments tailored toward depression, anxiety, or both.

Strikingly, SFR mice treated with fluoxetine during adolescence exhibit an anxiogenic phenotype in the EPM, an observation also made in other studies^22^,^23^. The reason for this effect is still unclear, especially since no such anxiogenic effects were detected in another test of anxiety-like behavior, the Light/Dark exploration (see Fig. 2c). It is possible that EPM exposure is a stronger stressor compared with exposure to the Light/Dark box and that this accounts for the observed anxiogenic effects. Yet, compared to fluoxetine mono-treatment, adolescent fluxetine/TSA co-treatment elicits anxiolytic effects, an effect not observed for TSA treatment alone. These findings suggest that reduced HDAC activity affects epigenetic and behavioral phenotypes only under conditions of increased serotonergic signaling, a possibility (also supported by results obtained from fluoxetine-treated IMS mice that were depleted of 5-HT (see Fig. 1)) that requires further investigation.

Finally, many of the epigenetic responses observed after adolescent fluoxetine/HDACi co-treatment were also observed in SFR mice treated in adulthood. Enrichment of acetylated H4K12 on Bdnf promotor 3 was elevated in MS-275 and TSA-co-treated mice, and both HDACis led to significantly increased density of Pol II at this promotor, an effect not seen with fluoxetine treatment alone. However, although all treatments elevated the levels of Bdnf transcript variant 3, only fluoxetine/TSA co-treatment raised these levels significantly.

In contrast to the lack of effect of adolescent fluoxetine alone on the behavior in the FST, adult fluoxetine treatment significantly improved the behavior of SFR Balb/c mice in this test. The failure of co-treatment with MS-275 in adulthood to further enhance the effect of fluoxetine is therefore likely due to a ceiling effect of fluoxetine treatment alone. However, adult fluoxetine treatment did not improve the behavior of SFR mice in tests of anxiety, and similar to results obtained after adolescent fluoxetine/HDACi co-treatment, TSA (but not MS-275) co-treatment elicited anxiolytic effects in the EPM and L/D test. In view of these findings it is worth noting that both adolescent and adult fluoxetine/TSA co-treatments led to anxiolytic effects and, as discussed above, while Bdnf transcript variant 3 mRNA is increased after this treatment, additional changes in gene expression may account for this effect.

In conclusion, this study illustrates that a greater understanding of the epigenetic mechanisms underlying antidepressant efficacy may guide the development of novel treatments for mood disorders. The data obtained from an animal model with robust responsiveness to fluoxetine treatment point to HDAC inhibitors as a powerful adjuvant to fluoxetine treatment not only for adolescent subjects with minimal response to fluoxetine mono-treatment and risk of suicide (see ref. 29), but also for adult SSRI non-responders. Since the combination of fluoxetine and valproic acid (which is an HDAC inhibitor^30^) is already used in the clinical practice, a systematic investigation of the efficacy of this co-treatment could give valuable insight into the clinical relevance suggested by the present study.

## Methods

### Animals

Balb/cJ mice were house in a temperature and light-controlled barrier facility with free access to food and water. All experiments involving the animals were performed in accordance with the National Institutes of Health Guide for the Care and Use of Laboratory Animals and approved by the Institutional Animal Care and Use Committees at Columbia University and the New York State Psychiatric Institute.

### Infant Maternal Separation (IMS)

Pups exposed to the IMS paradigm were separated from their mothers daily for three hours (from 1:00 to 4:00 PM), starting at postnatal age P2 and ending at P15 as previously described^13^. They were weaned at P28 and group-housed by sex. Other pups were left undisturbed with their mothers and were also weaned at P28. These mice are referred to as standard-facility reared (SFR). Housing and husbandry conditions were identical for SFR and IMS mice.

### Drug treatments

Drugs were administered to mice (males and females) starting at P35 and ending at P59 (adolescent treatment) or starting at P60 and ending at P84 (adult treatment). All drugs were administered via the drinking water. Drug intake was monitored daily and drugs were replenished every 48 hours. Mice were treated with fluoxetine (12–16 mg/kg/day), MS-275 (15 μM/day), sodium butyrate (NaB, 0.6 g/kg/day), or trichostatin A (TSA; 2.5 mg/kg/day). IMS mice only received fluoxetine. SFR mice received either fluoxetine alone or were co-treated with one of the three HDAC inhibitors. TSA was purchased from Santa Cruz (Santa Cruz, CA). All other drugs were purchased from Sigma (Sigma Aldrich, St. Louis, MO).

Some fluoxetine-treated IMS mice were co-treated with para-chlorophenylalanin (pCPA; Sigma). During the first 5 days of treatment, pCPA (300 mg/kg) was administered twice intraperitoneally. A previous study from this laboratory showed that this treatment leads to an 80% reduction 5-HT/5-HIAA levels^31^. To maintain reduced 5-HT levels, mice continued to receive an injection of pCPA every 24 hours until the end of fluoxetine treatment. Other fluoxetine-treated IMS mice were co-treated with the TrkB inhibitor Ana-12 (Sigma; 1 mg/kg/day in drinking water).

### Behavioral tests

Male and female mice were first tested in the Elevated Plus Maze (EPM). They were placed into the center of the maze and allowed to explore the maze for 5 min. The times spend in open arms and the number of open and closed arm crossings were recorded. Two days later, they were examined in the Light/Dark exploration test (L/D test). Mice were placed into the light compartment facing the entrance into the dark compartment and were allowed to explore the test box for 10 min. During this time, the number of L/D crosses and the total time spend in the light compartment was recorded. One week after the L/D test, mice were tested in a modified version of the Forced Swim Test (FST), a 6-min exposure on day 1 followed by another 6-min exposure 24 hours later. The number of passive episodes and their duration (in sec) during the last 4 min of the second exposure were compared between the different treatment groups. In all behavioral tests no significance differences were found for treatment responses of males and females. Hence, data obtained from both sexes were combined in the analysis.

### RNA extraction and real-time RT-PCR

RNA (10 μg), extracted via guanidine/cesium chloride ultracentrifugation from dissected forebrain neocortex of male and female mice, was reverse transcribed as previously described^14^. cDNA was amplified using the iQ Real-Time PCR detection system (Bio-Rad, Hercules, CA) and CYBR Green (Bio-Rad) using the Bdnf transcript variants 1 to 3 primer sequences published by Tsankova et al^32^. Cycle thresholds (Ct) of amplifications (normalized using Ct values obtained for amplification of β-actin) were expressed as 1/2^ΔCt^.

### Chromatin Immunoprecipitations (ChIP)

ChIP experiments were performed on fixed (1% paraformaldehyde) and sonicated forebrain neocortical tissues of male and female mice as previously described^14^. ChIP-grade anti-acH4K12 (Millipore, Temecula, CA) and anti-RNA Polymerase II CTD repeat YSPTSPS (Abcam, Inc., Cambridge, MA) antibodies were used in conjunction with protein A magnetic beads (Millipore). Immunoprecipitated DNA and a serial dilution of input DNA were analyzed by SYBR-Green real-time PCR using the Bdnf promotor-specific primer sequences previously published^32^. Cycle thresholds of PCR amplifications were normalized to Ct values obtained for 0.1% (acH4K12) or 1% (Pol II) input DNA and expressed as 1/2^ΔCt^.

## Author Contributions

C.S. designed and executed the experiments, analyzed the data, and prepared the manuscript.

## Supplementary Material

Supplementary Information Supplementary Data
