# Supplementary material for: An HDAC-dependent epigenetic mechanism that enhances the efficacy of the antidepressant drug fluoxetine
Source: Sci Rep. 2015 Feb 2;5:8171. doi: 10.1038/srep08171 (PMC4313090; doi:10.1038/srep08171)
Supplement: Supplementary Information — Supplementary Data [file srep08171-s1.pdf]

An HDAC-dependent epigenetic mechanism that enhances the efficacy of the antidepressant drug fluoxetine

C. Schmauss[1,2*]

[1]Department of Psychiatry, Columbia University and [2] Division of Molecular Therapeutics, New York State Psychiatric Institute, New York, NY 10032

SUPPLEMENTARY MATERIAL

<u>Supplementary Fig. 1</u> ***The effects of adolescent Ana-12 treatment on the emotional behavior of SFR mice.***

<u>Supplementary Fig. 2.</u> ***The effects of HDAC inhibitors alone on the emotional behavior of SFR mice.***

<u>Supplementary Fig. 3.</u> ***The effects of HDAC inhibitors alone on acH4K12 and Pol II enrichment at Bdnf promoters 1 to 3.***

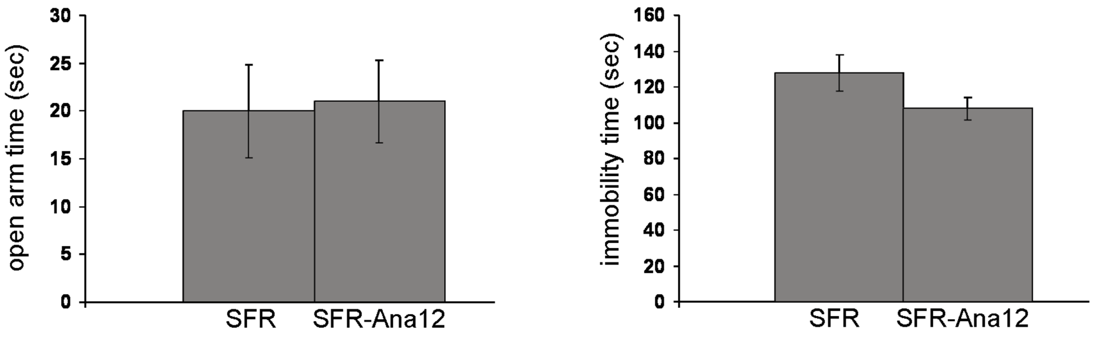

Supplementary Fig. 1. ***The effects of adolescent Ana-12 treatment on the emotional behavior of SFR mice.*** SFR mice treated during adolescence with Ana-12 exhibited unaltered behavior in the EPM (left) and a moderate decrease in immobility in the FST (right) that did not reach significance compared with non-treated SFR mice ($p=0.06$; Student's *t* test). Data are mean ± sem of 9 animals/group (5 males and 4 females).

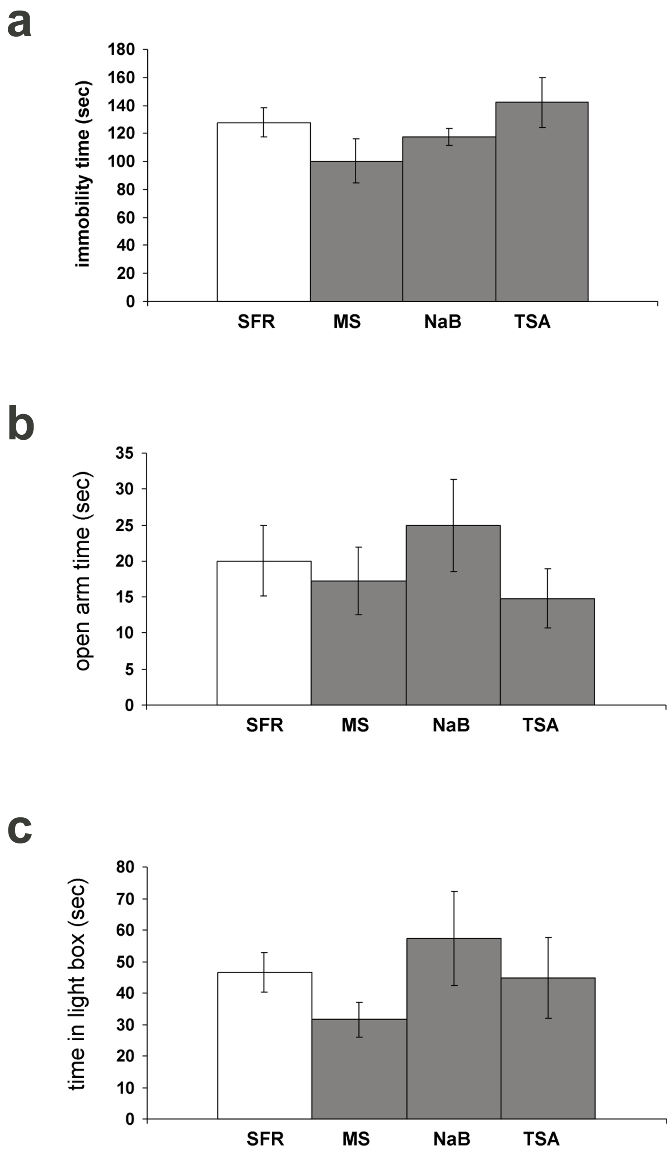

Supplementary Fig. 2. ***The effects of HDAC inhibitors alone on the emotional behavior of SFR mice.*** SFR mice treated during adolescence only with HDAC inhibitors exhibited unaltered behavior in (**a**) the FST (ANOVA, $F_{(3,26)}=1.704$, $p=0.19$)), (**b**) the EPM (ANOVA $F_{(3,30)}=0.5224$, $p=0.67$) and (**c**), the Light/Dark exploration (ANOVA, $F_{(3,25)}=1.41$, $p=0.266$). Data are mean ± sem of 6 to 8 animals/group (equal number of males and females).

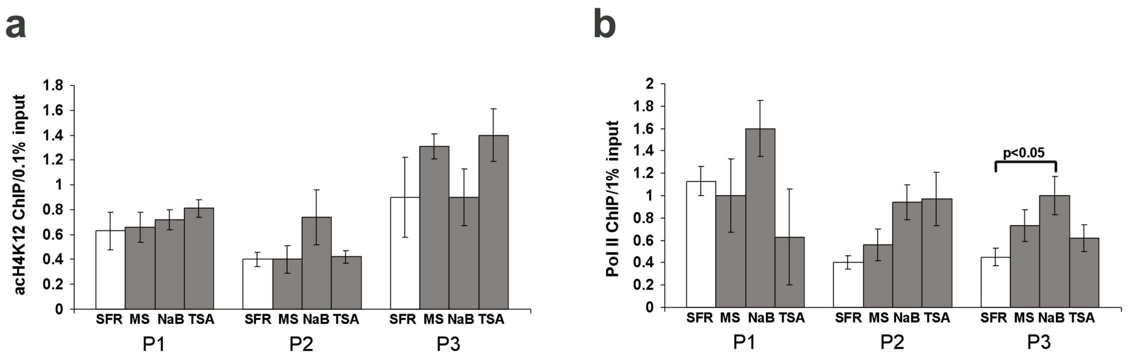

Supplementary Fig. 3. ***The effects of HDAC inhibitors alone on acH4K12 and Pol II enrichment at Bdnf promoters 1 to 3.*** **(a)** In SFR mice treated during adolescence only with HDAC inhibitors, neither HDAC inhibitor alone significantly increased acH4K12 levels at Bdnf promoters 1 to 3 (ANOVA, P1: $F_{(3,22)}$=0.6018, p=0.6; P2: $F_{(3,20)}$=3.65, p=0.04; P3: $F_{(3,20)}$=0.42, p=0.74)). **(b**) Pol II densities at Bdnf promotor 1 did not significantly differ (ANOVA, P1: $F_{(3,20)}$=0.7132, p=0.6) and, although HDAC inhibitor-treated SFR mice exhibited higher levels of Pol II at Bdnf promoter 2, these differences did not reach significance (ANOVA, P2: $F_{(3,20)}$=3.06, p=0.06)). HDAC inhibitor-treated SFR mice also exhibited higher levels of Pol II at Bdnf promoter 3 (ANOVA, P3: $F_{(3,20)}$=4.84, p=0.014)), but significantly higher levels of Pol II were only detected in NaB-treated mice.
